# Creating a Knowledge Translation Platform: nine lessons from the Zambia Forum for Health Research

**DOI:** 10.1186/1478-4505-10-31

**Published:** 2012-10-03

**Authors:** Joseph M Kasonde, Sandy Campbell

**Affiliations:** 1 Minister of Health, Ministry of Health Government of the Republic of Zambia, Ndeke House, PO BOX 30205, Lusaka, Zambia; 2 Knowledge Translation Specialist, Berkeley, CA, USA

**Keywords:** Knowledge translation platform, Networking, Zambia, ZAMFOHR, Synthesis, Brokering, Communications, Capacity building

## Abstract

The concept of the Knowledge Translation Platform (KTP) provides cohesion and leadership for national–level knowledge translation efforts. In this review, we discuss nine key lessons documenting the experience of the Zambia Forum for Health Research, primarily to inform and exchange experience with the growing community of African KTPs. Lessons from ZAMFOHR’s organizational development include the necessity of selecting a multi-stakeholder and -sectoral Board of Directors; performing comprehensive situation analyses to understand not only the prevailing research-and-policy dynamics but a precise operational niche; and selecting a leader that bridges the worlds of research and policy. Programmatic lessons include focusing on building the capacity of both policy-makers and researchers; building a database of local evidence and national-level actors involved in research and policy; and catalyzing work in particular issue areas by identifying leaders from the research community, creating policy-maker demand for research evidence, and fostering the next generation by mentoring both up-and-coming researchers and policy–makers. Ultimately, ZAMFOHR’s experience shows that an African KTP must pay significant attention to its organizational details. A KTP must also invest in the skill base of the wider community and, more importantly, of its own staff. Given the very real deficit of research-support skills in most low-income countries – in synthesis, in communications, in brokering, in training – a KTP must spend significant time and resources in building these types of in-house expertise. And lastly, the role of networking cannot be underestimated. As a fully-networked KTP, ZAMFOHR has benefited from the innovations of other KTPs, from funding opportunities and partnerships, and from invaluable technical support from both African and northern colleagues.

## Background

Since its emergence at the 2004 Ministerial Summit in Mexico, the concept of knowledge translation (KT) has become a leading approach in narrowing the gulf between research and policy. Often misunderstood as a technique to transfer research findings directly to policy, KT is more properly imagined as a dynamic set of approaches connecting research and policy processes.^a^ Evidence-informed policy is as much a goal of KT as policy-informed research, signaling profound changes in how policy is developed *and* in how research evidence is created.

To forge this cycle of policy-informed research leading to evidence-informed policy, KT isolates several important moments within this cycle. As KT is above all a social process, it focuses on building trust and dialogue among researchers, policy-makers and other research users [1-5]. Several recent innovations illustrate this social nature of KT, including the deliberations central to the development of evidence-informed policy briefs and to the creation of Rapid Responses, where researchers respond to policy-maker demand with a tailored synthesis of research evidence. As much as it seeks to open up the policy process by advancing evidence as a tailored or demanded input, KT also seeks to reform the research process – principally by trying to align research topics with policy needs (as opposed to, for instance, the needs or desires of funders). This has been done through, for instance, priority-setting exercises, where multiple stakeholders convene and use tested methods to deliberate, weigh, balance and rank competing priorities in health research. And lastly, KT efforts have mapped the research and policy communities to better identify the range of stakeholders and the local “evidence” (from the peer-reviewed to the grey) that is then stored in widely accessible databases.

While in some low- and middle-income contexts there are individual projects and efforts focused on each of the above activities, in recent years the concept of the Knowledge Translation Platform (KTP) has emerged to add some cohesion to these efforts [6-10]. A KTP is, typically, a national- or state-level entity designed to create and nurture links among researchers, policy-makers and other research-users; these links draw the research and policy communities closer together to ultimately create cycles of policy-informed evidence and evidence-informed policy. KTPs are ideally led by trustworthy, highly connected and credible experts, intermediaries who excel in various different fields, including evidence gathering, critical appraisal, facilitation, communication and networking. They almost certainly require experience – and command respect – in the worlds of both research and policy.

As an organization, a KTP may take several different forms. It may be a virtual, web-based entity; it may be a network that forms around a particular issue (e.g. obesity, mental health) or an event (e.g. World AIDS Day); or it may have conventional office premises. Where the KTP locates itself is a critical variable in its organization and operations – whether as part of government, a parastatal, a university, or as a member of civil society. Each of these positions comes with a set of advantages and drawbacks. For instance, as a civil society organization, a KTP may rely upon its neutrality and independence to successfully broker among different stakeholders; yet as an independent entity it may suffer from an uncertain or shifting funding base. As part of government (e.g. a unit within the Ministry of Health), a KTP may capitalize upon its proximity to the policy-making process to stoke demand for evidence or to strengthen the capacity of policy–makers to access, assess, adapt and apply research evidence; yet its proximity may compromise the neutrality essential to science in general and to KT in particular.

In terms of the services a KTP may offer, these include:

• brokering or facilitating meetings among multiple stakeholders;

• identifying and documenting local researchers, institutions, agencies and funders: *who’s who? who’s doing what? who’s funding what?*;

• creating databases of local research evidence;

• synthesizing and packaging research – tailoring and targeting – for a particular audience (ideally in response to stated policy needs);

• leading or contributing to efforts to shape the research agenda (e.g. through priority-setting exercises or gap analyses);

• strengthening the capacity of researchers (e.g. to understand the policy process; to pursue KT-informed activities), research-users (e.g. sensitizing the media to particular research findings), and policy-makers (e.g. from increasing their demand for evidence to building their abilities to access, assess, adapt and apply research evidence);

• undertaking advocacy to disseminate and support the use of research evidence; and

• amplifying the needs and input of various stakeholders on core research topics and key policy moments.

### Main text

Since independence, Zambia’s research and policy communities have evolved in very separate fashions. On one side, the research community has evolved within the silo of academia, with a primary influence seen in the strong vertical linkages with foreign funders. Research projects often have little communication with each other and tend to have a weak, peripheral connection with the national government. Findings from these projects often flee the country, appearing in global scientific journals without influencing or informing any local policy or practice.

The policy community, in turn, often formulates policy without consulting research evidence, and has few active connections with either independent researchers or the research community more broadly. They turn to many other trusted sources as inputs to their policy-making, and see little incentive in interacting with a research community often seen as “elite” – with researchers possessing multiple degrees from foreign universities, networked with specialists across the world, and devising public-health solutions that often do not reflect the policy-making reality.

While this context of two separate communities is hardly unique to Zambia [11], in 2005 explorations began to assess and address ways of narrowing the gap between these two communities. A planning team of stakeholders, including representatives from the national research and policy communities, and the international research community, came together to begin laying the groundwork for a Zambian KTP. In this paper, we describe the process behind both the organizational and programmatic development of the Zambia Forum for Health Research (ZAMFOHR) in order to highlight lessons that might inform the development of other KTPs across sub-Saharan Africa and beyond.

“Lessons” in this paper denotes conclusions the authors have drawn based upon numerous evaluative brainstorming sessions. In these sessions, we determined some key variables (organizational development, programming, KT innovations) and then analyzed how ZAMFOHR has responded to each in turn. These lessons are inherently qualitative reflections and are offered here in an evaluative spirit to inform the development of other KTPs. There are many limitations in arriving at these lessons, and there is a strong need for a research project to capture them in a much more rigorous fashion.^b^

### Organizational development

The first step in understanding the type of organization ZAMFOHR could be involved an extensive situation analysis. Starting with the recognition that the research community was deeply fragmented, ZAMFOHR’s planning team wanted to map out these fragments and arrive at a comprehensive picture of who was doing what, why, and where (and funded by whom). A local senior health-sector consultant was commissioned to execute several scoping surveys, comprised chiefly of key informant interviews at domestic research institutions to document details, collect papers, strategic plans, project profiles, and also to determine the degree of policy-maker involvement in their work. This simple act of databasing had never been done before, and allowed the planning team to start identifying and assessing some of the core issues within the research community – for instance, the systemic lack of incentives for researchers to collaborate or at the very least share information with each other, and the often corrosive spirit of competition among researchers for scarce research funds. Further analysis of these scoping studies revealed the routine lack of policy-maker involvement in research projects, and a significant number of final reports that had not been published or disseminated.

The planning team then turned to the policy community, and conducted some key informant interviews, primarily with Ministry of Health stakeholders. Designed again to assess the relationship between the research and policy communities, these interviews revealed a consistent distrust of researchers, a perception that researchers were playing a “very different game,” yet a consistent recognition that there had to be new ways not yet explored to bring together these two communities.

Understanding these intra-community dynamics – including institutional and individual conflicts, and a deeper awareness of power relations and how things really work – led to the next step in the process. This saw the planning team identify 30 different stakeholders from the research and policy communities to participate in deliberations on how (or even if) a KTP could serve their needs. The first such dialogue was convened in April 2006, and saw stakeholders discussing:

• why was there a disconnection between the research and policy communities?

• what specific activities could lead to more cohesion between them?

• what could a new organization like ZAMFOHR offer that existing organizations could not?

• where should ZAMFOHR locate itself? As an NGO? As a parastatal? As part of the Ministry of Health?

• given the scarce funding environment, how could an entity like ZAMFOHR find sustainable funding for its operations *and* not reduce the overall pot of funds available to health research?

**Figure 1:**
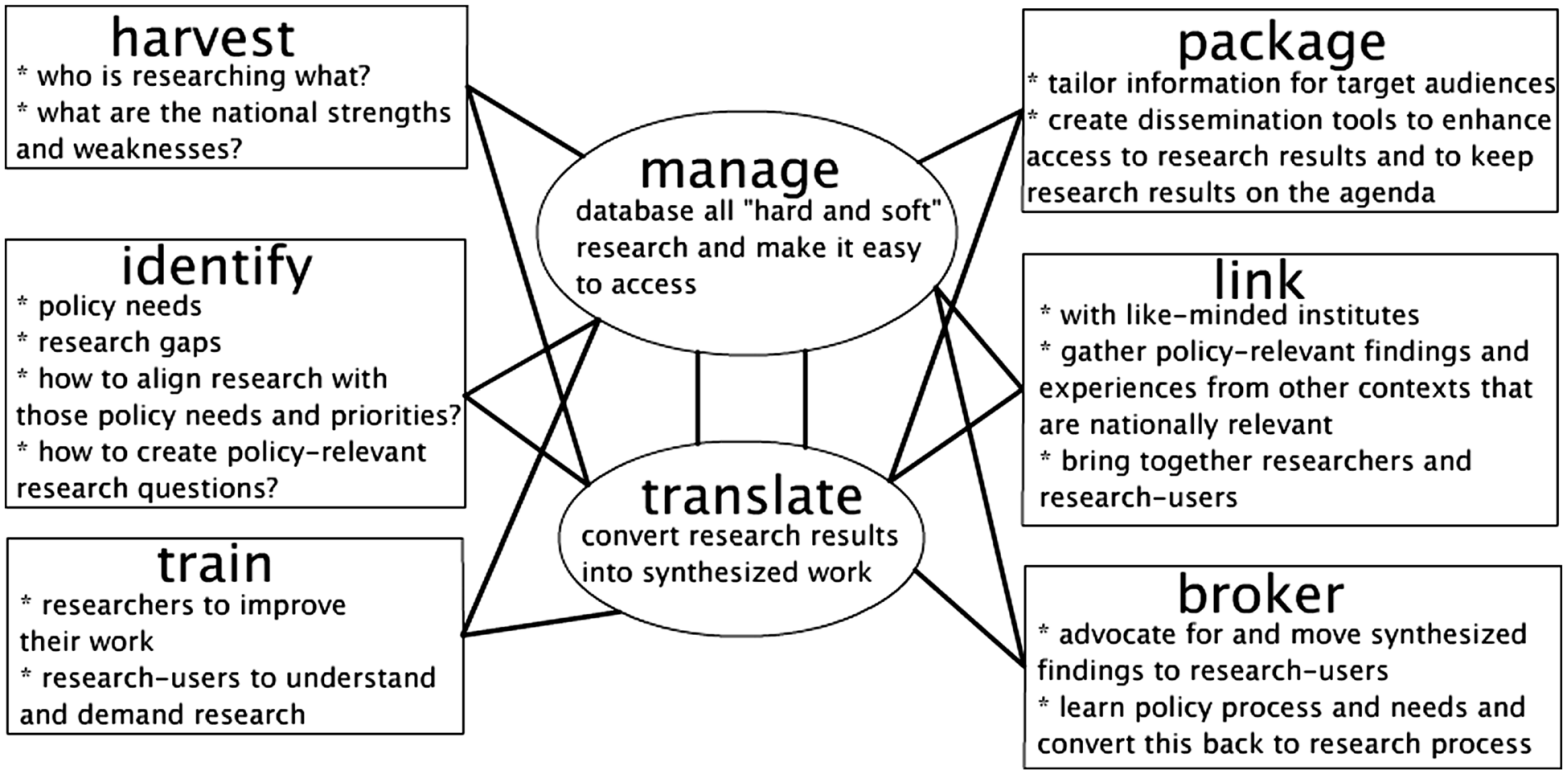
Source: Strategic Plan of the Zambia Forum for Health Research 2006-2010.

A second stakeholder meeting, in June 2006, used the results of the first dialogue as a starting point and began brainstorming a Strategic Plan for ZAMFOHR. With a vision that foresaw the creation of “*coordinated and responsive health research and evidence,”* ZAMFOHR ultimately determined five primary objectives for its first five-year period (2006–2010) (Figure 1):

• harvesting and harmonizing research – building a database of locally produced evidence

• identifying research needs of stakeholders on specific issues and facilitating priority setting among researchers and research users

• translating knowledge and promoting its use by stakeholders.

• facilitating linkages and networking among researchers, users and like-minded institutions locally, regionally and globally

• becoming a resource centre of information, providing access to information and offering broad capacity building initiatives.

#### Lesson 1

In creating the organization with a Strategic Plan endorsed by a range of different stakeholders, a central innovation was assembling a Board of Directors from across the research and policy communities. This Board was then given wide powers of oversight on the development of the Strategic Plan, as well as constant reflection on ZAMFOHR’s initial set of activities. Board members included senior individuals from research institutions, the director of public health and research at the Ministry of Health, the executive director of the Ministry’s major parastatal, the Tropical Disease Research Centre, and several senior representatives of the academic sector, all headed by a Chair with a background in research management. The composition of this multi-disciplinary, multi-sectoral Board was a critical early achievement of ZAMFOHR, and led directly to its buy-in among the community. Convincing local, national and even global stakeholders of ZAMFOHR’s function and utility is a phenomenon common to any new institution, but particularly acute when the field (KT) is itself relatively new and largely misunderstood.

#### Lesson 2

The situation analysis work that identified major stakeholders and the dynamics among them was a major step in illumining not only the terrain ZAMFOHR could occupy, but the stakeholders critical to its establishment. In 2005, the concept of situation analysis was relatively undeveloped, and there are more scientific tools to use today in doing such work. But the situation analysis identified two essential elements to ZAMFOHR’s initial functioning: the state of local evidence (its strengths and weaknesses, its actual location, and possible means for databasing it), and the stakeholders whose participation was essential in ZAMFOHR’s creation, not only in arriving at a responsive vision and mission, but in ensuring early buy-in from the community. A core aspect missing from this situation analysis work was a comprehensive understanding of the policy process in Zambia, including a more nuanced appreciation of power in the health sector.

#### Lesson 3

The choice of leadership for ZAMFOHR was complex, and another instrumental step. The leader ultimately selected and approved by the Board ^c^ had a unique combination of skills in both the research and policy communities, and carried sufficient respect among those communities to be an effective broker bringing both together.

#### Lesson 4

Funding issues have been a central concern for ZAMFOHR since its inception. While many funders acknowledge the importance of KT, few in fact fund it, leaving ZAMFOHR with limited options to fund its operations. There are research funders and there are development funders, but few who are willing to combine the two and see research support – i.e. all the things required to make research a viable policy input – as a poverty-reduction strategy in itself. Funders also have a great reluctance in offering core support, which is crucial for an organization that must maintain local offices to maintain visibility, generate confidence in its mission, broker meetings and convene dialogues.

ZAMFOHR’s reliance on external funding is a weakness in its organizational structure. KTPs must increasingly make the argument that KT is a core health-system activity and thus must be funded via structured domestic arrangements (e.g. attracting Ministry of Health funding or support) that – critically – do not impinge upon their neutrality.

### Programmatic development

Noting that this paper does not seek to assess the success or failures of ZAMFOHR’s programming, instead are a series of lessons distilled from its major programmatic activities (as reflected in the Strategic Plan above) over 2006–2010.

#### Lesson 5

Strengthening the capacity of researchers, policy-makers and other research-users is absolutely essential. It has been the most welcome activity of ZAMFOHR, though unfortunately also the most expensive. At the same time, there were significant consistency issues – with the training itself (i.e. lack of curricula, some trainers better than others, some able to do multiple courses but most able to offer only one, and, significantly, most hailing from the global north), but principally with the people receiving the training. It proved difficult to build a “skill baseline” among the research and policy community as the individuals receiving the training were always changing. Why were participants always so different? Likely ZAMFOHR’s training invitations were too broad, did not define narrowly the kind of people needed and thus encouraged a collective entitlement. This resulted in ZAMFOHR refining its training invitations:

• those invited were not allowed to send substitutes

• a Call for Participation went out for several training opportunities, with delegates needing to respond in precise details why they should be considered.

Transportation and per diem issues constantly plagued training sessions, where delegates insisted on financial remuneration for their time and thus dramatically increased the associated costs.

ZAMFOHR’s experience in capacity building leaves the conclusion that strengthening KT skills is critical, with a great deal remaining to be done. A KTP can perhaps best contribute to this by understanding the existing skill bases and what can be enhanced, and then offering (or supporting) regular, systematized courses in core skills tailored to the particular health-system context.

#### Lesson 6

Creating a database of local evidence (who’s doing what; who’s published what; who’s funding what) was, and remains, an enormous undertaking.^d^ It remains to be seen whether an NGO like ZAMFOHR is in fact best suited to do this kind of work, or whether it should instead be performed by the Ministry of Health or other government agency. Convincing institutions to contribute their findings and literature is an ongoing challenge, as they have few incentives for doing so and may perceive it as a violation of their intellectual property rights. Secondly, Zambia has always had significant connectivity issues, and thus the ability to upload and download documents is strongly impaired. The development of this database has correspondingly suffered as users cannot reliably access it, provide feedback to improve it, and make active contributions to it.

#### Lesson 7

Zambia’s wider operating framework has strongly influenced ZAMFOHR’s functionality. There are relatively few skilled individuals capable of databasing, of offering training courses, of facilitating dialogues, of conducting priority setting exercises, and on. This has seen the ED performing many of these tasks, resulting in severe over-stretching. Succession planning – identifying new leadership for ZAMFOHR – has also been extremely difficult given the few individuals who truly bridge both the research and policy communities. As with many research institutions in low-income settings, this dependence on one individual has created a great vulnerability for the institution’s long-term prospects.

### KTP innovations

Despite the challenges outlined in lessons 5–7, over 2006–2010, ZAMFOHR was able to experiment with different sets of KT activities and arrive at some important innovations. These include:

• creating Research-to-Action Groups (RAGs). These groups work to focus KT activities on a specific issue, which to date have included mental health, reproductive health, and human resources for health. These RAGs have a decentralized leadership – i.e. they’re led by someone other than ZAMFOHR’s ED – and work to identify all the relevant stakeholders and dynamics within that issue. Critically, they serve to identify the up-and-coming individuals within the issue domain, be they policy-makers or researchers, with the leadership then serving to mentor them as need be. RAGs have also led policy briefs and dialogues (see below), and were instrumental in identifying KT Fellows. These Fellows have become a leading resource on issue-specific topics, and in KT more generally. This has seen them offer training, lead the development of the RAGs, identify young researchers for mentoring, and may well see them participate in overseas training (particularly through ZAMFOHR’s global partnerships – see Lesson 9 below).

• leading policy briefs and dialogues. The RAGs contributed directly to the creation of evidence-informed policy briefs and dialogues.^e^ These have been enormously successful KT moments, where multiple stakeholders identify a policy priority, assemble a list of evidence-informed policy options, deliberate as a group on those options, and then assist policy–makers in developing and implementing a formal policy response. The reproductive health and mental health RAGs have led briefs/dialogues of particular note.^f^

• creating a rapid response service (RRS). This service is designed to encourage policy-makers to ask a specific question, with ZAMFOHR then turning around in a set timeframe and answering the question with the best available research evidence. While this service did not officially begin operating between 2006 and 2010, much of ZAMFOHR’s experience during this time suggested the necessity of it. In 2011, through technical and financial support from the Evidence-Informed Policy Networks (EVIPNet) of WHO, ZAMFOHR began preparing for the service. Staff were trained in January 2012 on running the RRS, covering all details from its organization to its precise functions. It is currently completing the first two Responses, and is expected to become a core part of ZAMFOHR’s mandate by the end of 2012.

#### Lesson 8

Policy briefs and dialogues are just beginning to show their value in bringing together the research and policy communities. Above all, their strength has been in creating open spaces for discussion among different groups of stakeholders – whether that discussion features research evidence or not. Importantly, however, there remains a great distance between preparing the brief and then implementing any of the available options. While merely a facilitator to date, ZAMFOHR must now decide upon the role it might play in solving some of the clear challenges in actually implementing evidence-informed policy. How might ZAMFOHR start to position itself to assist the government with these implementation challenges? Should ZAMFOHR participate in actual policy implementation? And if so – how?

#### Lesson 9

Throughout ZAMFOHR’s operations, international partnerships have been essential. ZAMFOHR’s membership in the network supported by EVIPNet has allowed it to benefit from the innovations and experience of like-minded KTPs in other African countries. Funds and technical support through the Supporting the Use of Evidence in African Health Systems (SURE) project (funded by the European Commission), and the Alliance for Health Policy and Systems Research have been integral to the methodological development of these KT tools. ZAMFOHR’s long strategic partnership with the Canadian Coalition for Global Health Research (CCGHR) has underscored all of ZAMFOHR’s activities, particularly in delivering training, in providing Canadian interns to assist in executing ZAMFOHR’s mandate, in fundraising, in databasing, and in providing general overall technical support.^g^ Lastly, Canada’s International Development Research Centre (IDRC) has been the catalyst for many of these activities, with its initial seed funding in 2006 sprouting into many of the above-mentioned innovations.

## Conclusions

As the ZAMFOHR experience has shown, a KTP is indeed a viable concept. While every context may require different forms and different services from a KTP, in Zambia its shape and functions arose through routine and wide stakeholder consultation and involvement. While uncertainty over its funding base has been, and continues to be, a persistent feature, many of ZAMFOHR’s core tasks – from capacity strengthening to dialogues – have become more or less institutionalized, with the Ministry of Health becoming a routine demander of its services.

Lesson 9 above discusses some of the global partnerships essential to ZAMFOHR’s success, but of particular note here are the alliances ZAMFOHR has with like-minded KTPs – especially those in Uganda (REACH-Policy) and Cameroon (the Centre for the Development of Best Practices in Health). African KTPs participate in an active network (funded by IDRC and the EC and led by EVIPNet and SURE), which has been critical in testing and diffusing innovations. The success of the policy brief and dialogue model in Zambia is a direct testament to this networking: all African KTPs in the EVIPNet and SURE network have participated in various methodological workshops focused on the brief and dialogue, and then given each other technical support in developing local policy briefs and dialogues. Noting the same process is underway for the Rapid Response Services (with Ugandan trainers from REACH-Policy currently supporting ZAMFOHR and a KTP in Burkina Faso), this connection with like-African experiences is essential, with each able to build on the shoulders of others.

In summary, ZAMFOHR’s experience from idea in 2005 to active NGO in 2012 illustrates three elements essential to an African KTP. First, a KTP must pay routine and significant attention to its organizational details: *form follows function*. While there may have been some early activities ZAMFOHR could have pursued to prove its value, it chose instead to follow the longer path of identifying stakeholders, studying research-policy relationships, selecting a well-suited leader, determining its organizational status, and creating a dynamic core of individuals across disciplines. Second, a KTP must be prepared to invest in the skill base – of the wider community (e.g. training policy–makers to demand research evidence) and, more importantly, of its own staff. Given the very real deficit of research-support skills in most low-income countries – in synthesis, in communications, in brokering, in training – a KTP must spend significant time and resources in building these types of in-house expertise. And lastly, the role of networking cannot be underestimated. As a fully-networked KTP, ZAMFOHR has benefited from the innovations of other KTPs, from funding opportunities and partnerships, and from invaluable technical support from both African and northern colleagues.

## Endnotes

^a^While noting CIHR’s valuable and oft-quoted definition of KT as “a dynamic and iterative process that includes synthesis, dissemination, exchange and ethically-sound application of knowledge…” [12] in this paper we understand KT more broadly as a *dynamic, context-shaped process creating cycles of evidence-informed policy and policy-informed evidence*. See Graham and Tetroe (2009) for full KT definitions.

^b^Note that ZAMFOHR is participating in the monitoring and evaluation (M&E) work currently conducted by the McMaster Knowledge Translation team based at McMaster University in Hamilton, Canada. This work focuses on the outputs, outcome and impact of applying KT tools to influence policies (such as policy briefs and dialogues). In addition, this KT team is capturing lessons learned from evidence-to-policy initiatives in several countries through structured reflection.

^c^The first author of this paper.

^d^To see ZAMFOHR’s evolving database, see http://www.zamfohr.org.

^e^For more on the policy brief and dialogue as a KT intervention, see the SUPPORT tools: http://www.support-collaboration.org/supporttool.htm and the SURE Guides: http://www.who.int/evidence/sure/guides/en/index.html.

^f^To see the mental health RAG’s policy brief, visit http://www.who.int/rpc/evipnet/MentalhealthZambia.pdf.

^g^The CCGHR-ZAMFOHR Internship program – while currently on hiatus pending further funding – has been a highly evaluated program. This has worked in particular to build ZAMFOHR’s staff skills (e.g. in databasing, research methods, writing support, and proposal development) while also giving important African experience to the next generation of Canadian researchers.

## Competing interests

Both authors declare they have no competing interests. Neither they nor the institutions with which they work will benefit from the publication of this article. They were both instrumentally involved in the creation of the institution discussed here, and for five years the first author was paid by the institution. He stepped down from this position in September 2011 upon appointment as Minister of Health. The second author has never received payment from this institution, though has been involved in raising funds for it.

## Authors’ contributions

JK and SC contributed equally to the development of this paper. JK provided particular contributions to the intellectual content of the paper. JK wrote the first draft, which served as a focus prompt for further brainstorming between the authors. SC authored all ensuing drafts. No financial support was received for the writing of this paper. Both authors read and approved the final manuscript.

## Author information

JK was the Executive Director of ZAMFOHR from its inception in 2005 until his appointment as Zambia’s Minister of Health in 2011. As an employee of Canada’s International Development Research Centre (IDRC), SC contributed intellectual support to the establishment and early operations of ZAMFOHR. IDRC provided start-up funds to the organization. From 2008 to the present, SC has provided technical support to ZAMFOHR (leading capacity strengthening workshops, consulting on its organizational management) as a consultant for both the Canadian Coalition for Global Health Research (CCGHR) and the WHO’s Evidence-Informed Policy Network (EVIPNet).
